# Supplementary material for: Comparative genomics provides new insights into the diversity, physiology, and sexuality of the only industrially exploited tremellomycete: Phaffia rhodozyma
Source: BMC Genomics. 2016 Nov 9;17:901. doi: 10.1186/s12864-016-3244-7 (PMC5103461; doi:10.1186/s12864-016-3244-7)
Supplement: Additional file 6: — List of orphan genes with links to PFAM (related to Additional file 1: Table S1). (ZIP 1428 kb) [file 12864_2016_3244_MOESM6_ESM.zip › BLAST_HTML_FTR/G02106_P.html]

BLAST Search Results


```
BLASTP 2.2.27+


Reference:
Stephen F. Altschul, Thomas L. Madden, Alejandro A. Schäffer,
Jinghui Zhang, Zheng Zhang, Webb Miller, and David J. Lipman (1997),
"Gapped BLAST and PSI-BLAST: a new generation of protein database
search programs", Nucleic Acids Res. 25:3389-3402.


Reference for
composition-based statistics:
Alejandro A. Schäffer, L. Aravind, Thomas L. Madden, Sergei
Shavirin, John L. Spouge, Yuri I. Wolf, Eugene V. Koonin, and
Stephen F. Altschul (2001), "Improving the accuracy of PSI-BLAST
protein database searches with composition-based statistics and
other refinements", Nucleic Acids Res. 29:2994-3005.


Database: nr
           71,551,133 sequences; 26,053,659,533 total letters


Query= G02106_P

Length=556
                                                                      Score     E
Sequences producing significant alignments:                          (Bits)  Value

emb|CED82171.1|  hypothetical protein [Xanthophyllomyces dendrorh...   640    0.0  
emb|CED82170.1|  hypothetical protein [Xanthophyllomyces dendrorh...   254    3e-78


 >emb|CED82171.1| hypothetical protein [Xanthophyllomyces dendrorhous]
Length=422

 Score =  640 bits (1650),  Expect = 0.0, Method: Compositional matrix adjust.
 Identities = 359/413 (87%), Positives = 363/413 (88%), Gaps = 42/413 (10%)

Query  1    MASSKLQSNSRFAKRPPSSSTASDSSLPVQSSRRDRDRRSGEVKSLPTSVSVKVNSGDNS  60
            MASSKLQSNSRFAKRPPSSSTASDSSLPVQSSRRDRDRRSGEVKSLPTSVSVKVNSGDNS
Sbjct  7    MASSKLQSNSRFAKRPPSSSTASDSSLPVQSSRRDRDRRSGEVKSLPTSVSVKVNSGDNS  66

Query  61   RKPRTHDLRNETNQNKQLNNQRHQRQEGM-------------------------------  89
            RKPRTHDLRNETNQNKQLNNQRHQRQEGM                               
Sbjct  67   RKPRTHDLRNETNQNKQLNNQRHQRQEGMVRDKSSGTRPAILRETQTPRELASNKQNEGM  126

Query  90   -------QTGKKKPKRRKGNEKDTSPLADSEKRKEASMTLENLLDDMSKQAIRPMPQVHV  142
                   QTGKKKPKRRKGNEKDTSPLADSEKRKEASMTLENLLDDMSKQAIRPMPQVHV
Sbjct  127  IIRNDHQQTGKKKPKRRKGNEKDTSPLADSEKRKEASMTLENLLDDMSKQAIRPMPQVHV  186

Query  143  RTKSSQPQTHQNKPENKPDENKRRERNATLASSEGSRTIPGRSDLHRDNDHHQSIRSPRS  202
            RTKSSQPQTHQNKPENKPDENKRRERNATLASSEGSRTIPGRSD HRDNDHHQSIRSPRS
Sbjct  187  RTKSSQPQTHQNKPENKPDENKRRERNATLASSEGSRTIPGRSDRHRDNDHHQSIRSPRS  246

Query  203  LPDDSPSTNNHCRASIKENVSQKRSSNRPQRNSIRHEPTTLRRPSSDNSTSGHHALVGSP  262
            LPDDSPSTNNHCRASIKENVSQKRSSNRPQRNSIRHEPTTLRRPSSDNSTSGHHALVGSP
Sbjct  247  LPDDSPSTNNHCRASIKENVSQKRSSNRPQRNSIRHEPTTLRRPSSDNSTSGHHALVGSP  306

Query  263  EPEQNIKINTPQVKQLQPEHEFDPSQNGDFVPTKTPYVK----RDPGHTTIKKQFSINDI  318
            EPEQN KINTPQVKQLQPEHEFDPSQNGDFVPTKTPYVK    RDPGHTTIK+QFSINDI
Sbjct  307  EPEQNTKINTPQVKQLQPEHEFDPSQNGDFVPTKTPYVKIVCQRDPGHTTIKRQFSINDI  366

Query  319  LVSAIWTLPPTPPSLSHPEGQQSYQVTVKDPYDLGRALKMKSTPLWNRRANIE  371
            LVSAIWTLPPTPPSLSHPEGQQSYQVTVKDPYDLGRALKMKS+      A++E
Sbjct  367  LVSAIWTLPPTPPSLSHPEGQQSYQVTVKDPYDLGRALKMKSSSFRIISAHLE  419


>emb|CED82170.1| hypothetical protein [Xanthophyllomyces dendrorhous]
Length=135

 Score =  254 bits (649),  Expect = 3e-78, Method: Compositional matrix adjust.
 Identities = 121/121 (100%), Positives = 121/121 (100%), Gaps = 0/121 (0%)

Query  415  RAVNTTTRSLSASRLCYSTDLGRPAADTVLQGKLLVDVEYKTSKYGDKRTYASINIKRLK  474
            RAVNTTTRSLSASRLCYSTDLGRPAADTVLQGKLLVDVEYKTSKYGDKRTYASINIKRLK
Sbjct  6    RAVNTTTRSLSASRLCYSTDLGRPAADTVLQGKLLVDVEYKTSKYGDKRTYASINIKRLK  65

Query  475  QDKTEFDIFNIVAYGEEAVRYVREDLASAKSGSTVEVGVSLFSRREQLAAHIRNEKPFFI  534
            QDKTEFDIFNIVAYGEEAVRYVREDLASAKSGSTVEVGVSLFSRREQLAAHIRNEKPFFI
Sbjct  66   QDKTEFDIFNIVAYGEEAVRYVREDLASAKSGSTVEVGVSLFSRREQLAAHIRNEKPFFI  125

Query  535  H  535
            H
Sbjct  126  H  126


Lambda      K        H        a         alpha
   0.312    0.126    0.357    0.792     4.96 

Gapped
Lambda      K        H        a         alpha    sigma
   0.267   0.0410    0.140     1.90     42.6     43.6 

Effective search space used: 5956673114000


  Database: nr
    Posted date:  Sep 23, 2015 12:05 AM
  Number of letters in database: 26,053,659,533
  Number of sequences in database:  71,551,133


Matrix: BLOSUM62
Gap Penalties: Existence: 11, Extension: 1
Neighboring words threshold: 11
Window for multiple hits: 40
```
